# A new crystal modification of diammonium hydrogen phosphate, (NH_4_)_2_(HPO_4_)

**DOI:** 10.1107/S1600536810009839

**Published:** 2010-03-20

**Authors:** Peter C. Kunz, Corinna Wetzel, Bernhard Spingler

**Affiliations:** a Heirich-Heine-Universität Düsseldorf, Institut für Anorganische Chemie und Strukturchemie I, Universitätsstrasse 1, D-40225 Düsseldorf, Germany; b Anorganisch-Chemisches Institut, Universität Zürich-Irchel, Winterthurerstrasse 190, CH-8057 Zürich, Switzerland

## Abstract

The addition of hexa­fluorido­phosphate salts (ammonium, silver, thallium or potassium) is usually used to precipitate complex cations from aqueous solutions. It has long been known that PF_6_^−^ is sensitive towards hydrolysis under acidic conditions [Gebala & Jones (1969 ▶). *J. Inorg. Nucl. Chem.* **31**, 771–776; Plakhotnyk *et al.* (2005 ▶). *J. Fluorine Chem.* **126**, 27–31]. During the course of our investigation into coinage metal complexes of diphosphine ligands, we used ammonium hexa­fluorido­phosphate in order to crystallize [Ag(diphos­phine)_2_]PF_6_ complexes. From these solutions we always obtained needle-like crystals which turned out to be the title compound, 2NH_4_^+^·HPO_4_^2−^. It was received as the hydrolysis product of NH_4_PF_6_. The crystals are a new modification of diammonium hydrogen phosphate. In contrast to the previously published polymorph [Khan *et al.* (1972 ▶). *Acta Cryst.* B**28**, 2065–2069], *Z′* of the title compound is 2. In the new modification of the title compound, there are eight mol­ecules of (NH_4_)_2_(HPO_4_) in the unit cell. The structure consists of PO_3_OH and NH_4_ tetra­hedra, held together by O—H⋯O and N—H⋯O hydrogen bonds.

## Related literature

For the study of another crystal modification of the title compound, see: Khan *et al.* (1972 ▶). For the hydrolysis of hexa­fluorido­phosphates, see: Akbayeva *et al.* (2006 ▶); Deifel *et al.* (2008 ▶); Fernandez-Galan *et al.* (1994 ▶); Gebala & Jones (1969 ▶); Nikitenko *et al.* (2007 ▶); Plakhotnyk *et al.* (2005 ▶).

## Experimental

### 

#### Crystal data

2NH_4_^+^·HPO_4_^2−^
*M* *_r_* = 132.06
Monoclinic, 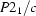

*a* = 11.2868 (3) Å
*b* = 15.3466 (4) Å
*c* = 6.41894 (19) Å
β = 90.795 (3)°
*V* = 1111.74 (5) Å^3^
*Z* = 8
Mo *K*α radiation
μ = 0.42 mm^−1^
*T* = 183 K
0.44 × 0.17 × 0.11 mm

#### Data collection

Oxford Xcalibur Ruby CCD diffractometer
Absorption correction: multi-scan *CrysAlis PRO* (Oxford Diffraction, 2009 ▶) *T*_min_ = 0.891, *T*_max_ = 0.955
22537 measured reflections
5384 independent reflections
4400 reflections with *I* > 2σ(*I*)
*R*_int_ = 0.033

#### Refinement

*R*[*F* ^2^ > 2σ(*F* ^2^)] = 0.048
*wR*(*F* ^2^) = 0.158
*S* = 1.21
5384 reflections
181 parameters
16 restraints
H atoms treated by a mixture of independent and constrained refinement
Δρ_max_ = 0.82 e Å^−3^
Δρ_min_ = −0.69 e Å^−3^

### 

Data collection: *CrysAlis PRO* (Oxford Diffraction, 2009 ▶); cell refinement: *CrysAlis PRO*; data reduction: *CrysAlis PRO*; program(s) used to solve structure: *SIR97* (Altomare *et al.*, 1999 ▶); program(s) used to refine structure: *SHELXL97* (Sheldrick, 2008 ▶); molecular graphics: *ORTEP-3* (Farrugia, 1997 ▶); software used to prepare material for publication: *SHELXL97* and *PLATON* (Spek, 2009 ▶).

## Supplementary Material

Crystal structure: contains datablocks global. DOI: 10.1107/S1600536810009839/br2137sup1.cif

Structure factors: contains datablocks I. DOI: 10.1107/S1600536810009839/br2137Isup2.hkl

Additional supplementary materials:  crystallographic information; 3D view; checkCIF report

## Figures and Tables

**Table 1:** Hydrogen-bond geometry (Å, °)

*D*—H⋯*A*	*D*—H	H⋯*A*	*D*⋯*A*	*D*—H⋯*A*
O1—H1⋯O7^i^	0.78 (4)	1.80 (4)	2.570 (2)	168 (4)
O5—H5⋯O8^ii^	0.85 (4)	1.79 (4)	2.632 (2)	170 (4)
N11—H11*A*⋯O4^iii^	0.86 (2)	1.89 (2)	2.747 (2)	175 (4)
N11—H11*B*⋯O8^i^	0.86 (2)	2.10 (2)	2.951 (2)	171 (3)
N11—H11*C*⋯O3^iv^	0.88 (2)	1.99 (2)	2.852 (3)	169 (3)
N11—H11*D*⋯O4^i^	0.88 (2)	2.01 (2)	2.870 (2)	167 (3)
N12—H12*A*⋯O6^v^	0.87 (2)	1.91 (2)	2.755 (2)	165 (3)
N12—H12*B*⋯O5^vi^	0.88 (2)	2.16 (2)	3.008 (3)	161 (3)
N12—H12*C*⋯O6^i^	0.87 (2)	1.99 (2)	2.827 (2)	161 (3)
N12—H12*D*⋯O2	0.88 (2)	1.88 (2)	2.754 (2)	175 (3)
N13—H13*A*⋯O3	0.86 (2)	1.92 (2)	2.773 (2)	172 (3)
N13—H13*B*⋯O2^vii^	0.86 (2)	1.96 (2)	2.822 (2)	175 (3)
N13—H13*C*⋯O6^v^	0.89 (2)	1.95 (2)	2.830 (2)	168 (3)
N13—H13*D*⋯O2^ii^	0.86 (2)	1.96 (2)	2.820 (2)	176 (3)
N14—H14*A*⋯O7	0.87 (2)	1.90 (2)	2.771 (2)	174 (3)
N14—H14*B*⋯O8^vii^	0.86 (2)	2.01 (2)	2.859 (2)	171 (3)
N14—H14*C*⋯O3^iv^	0.86 (2)	1.92 (2)	2.784 (2)	178 (3)
N14—H14*D*⋯O4	0.89 (2)	1.89 (2)	2.771 (2)	171 (3)

Symmetry codes: (i) 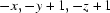; (ii) 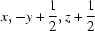; (iii) 
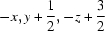
; (iv) 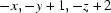; (v) 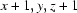; (vi) 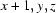; (vii) 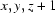.

**Fig. 1.**
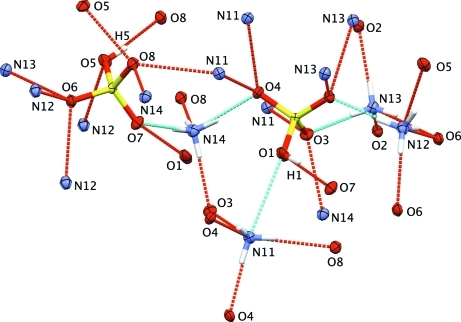
H-bonded network in the solid-state of the title compound. Displacement ellipsoids are drawn at a 50 % level, H-atoms are represented as capped sticks.

## supplementary crystallographic information

### Comment

The addition of hexafluorophosphate salts (ammonium, silver, thallium or potassium) is usually used to precipitate complex cations from aqueous solutions. It is long known, that PF_6_^-^ is sensitive towards hydrolysis under acidic conditions (Gebala and Jones 1969; Plakhotnyk, Ernst *et al.* 2005). In organic solvents the spatial hydrolysis to intermediate species as HF, POF_3_ and PO_2_F_2_^-^ is observed (Fernandez-Galan, Manzano *et al.* 1994; Akbayeva, Vaira *et al.* 2006; Nikitenko, Berthon *et al.* 2007) and under hydrothermal conditions this reaction is used for the formation of phosphate materials (Deifel, Holman *et al.* 2008).

During the course of our investigation into coinage metal complexes of diphosphine ligands we used ammonium hexafluorophosphate in order to crystallise [Ag(diphosphine)_2_]PF_6_ complexes. From these solutions we always obtained needle-like crystals which turned out to be the title compound (NH_4_)_2_(HPO_4_) as the product of hydrolysis of NH_4_PF_6_.

A modification of diammonium hydrogen phosphate is known with the cell parameters *a* = 11.043 (6), *b* = 6.700 (3), *c* = 8.031 (4), β = 113.42 (3)° and Z = 4.(Khan, Roux *et al.* 1972) In the new modification of the title compound reported here, there are eight molecules of (NH_4_)_2_(HPO_4_) in the unit cell. The structure consists of PO_4_ and NH_4_ tetrahedra, held together by O—H···O and N—H···O hydrogen bonds. These hydrogen bonds are more or less linear (169 ° < <(DHA) < 178 °). Of the four P-O bonds in both PO_4_ tetrahedra, one is longer than the remaining three, typical of a O_3_P(OH) group. The two different HPO_4_^-^ molecules (P1O_4_ and P2O_4_) are hydrogen bonded to ten and seven ammonium ions, respectively. In hydrogen phosphate P2, one NH_4_^+^ molecule is bound to O5 and O7, two to O8 and three to O6. In the other hydrogen phosphate, the three non-protonated O-atoms O2, O3 and O4 are bound to three NH_4_^+^ molecules each, whereas the protonated O1 is only bound to one NH_4_^+^ molecule. In the hydrogen phosphate P1 the hydroxyl group O1H1 forms a hydrogen bridge to O7 (*d* = 1.80 (4) Å) and in the hydrogen phosphate P2 the hydroxyl group O5H5 forms a hydrogen bridge to O8 (*d* = 1.79 (4) Å). The N—O distances around the four-coordinated ammonium ions N13 and N14 are within the range of 2.77 < *d* < 2.86 Å; The N···O distances around N12 fall within this range with the exception of N12···O5 which is significantly longer (*d*_N12O5_ = 3.007 Å). A very different picture is found around N11. Here, five neighbouring O-atoms are found, three of which are in a shorter distance (2. 74 < *d* < 2.87 Å) and two in a longer distance (*d*_N11O5_ = 2.951 Å and *d*_N1101_ = 3.048 Å). The fifth N···O contact may be a result of dynamic or static disorder of the ammonium ion or that each N atom in addition to three normal N—H···O bonds also formed one bifurcated bond. This is in contrast to the other modification, in which each ammonium ion has five N···O contacts smaller than 3.4 Å. Since Khan and Roux (Khan, Roux *et al.* 1972) only reported that they used "a commercially supplied crystalline sample", we cannot compare the crystallization conditions that lead to the two different crystal forms.

### Experimental

A suitable crystal was covered with oil (Infineum V8512, formerly known as Paratone N), mounted on top of a glass fibre and immediately transferred to the diffractometer. The final model was checked for higher symmetry with help of the program PLATON (Spek, 2009).

### Refinement

All hydrogen atoms were located by difference Fourier synthesis and refined with fixed individual displacement parameters [U(H) = 1.5Ueq(O)] at an O-H distance of 0.87 Å.

### Crystal data

**Table:**

2NH_4_^+^·HPO_4_^2^^−^	*F*(000) = 560
*M**_r_* = 132.06	*D*_x_ = 1.578 Mg m^−^^3^
Monoclinic, *P*2_1_/*c*	Mo *K*α radiation, λ = 0.7107 Å
Hall symbol: -P 2ybc	Cell parameters from 11185 reflections
*a* = 11.2868 (3) Å	θ = 2.7–37.6°
*b* = 15.3466 (4) Å	µ = 0.42 mm^−^^1^
*c* = 6.41894 (19) Å	*T* = 183 K
β = 90.795 (3)°	Needle, colourless
*V* = 1111.74 (5) Å^3^	0.44 × 0.17 × 0.11 mm
*Z* = 8	

### Data collection

**Table:**

Oxford Xcalibur Ruby CCD diffractometer	5384 independent reflections
Radiation source: Enhance (Mo) X-ray Source	4400 reflections with *I* > 2σ(*I*)
graphite	*R*_int_ = 0.033
Detector resolution: 10.4498 pixels mm^-1^	θ_max_ = 36.3°, θ_min_ = 2.7°
ω oscillation scan	*h* = −18→18
Absorption correction: multi-scan *CrysAlis PRO* (Oxford Diffraction, 2009)	*k* = −25→25
*T*_min_ = 0.891, *T*_max_ = 0.955	*l* = −9→10
22537 measured reflections	

### Refinement

**Table:**

Refinement on *F*^2^	Primary atom site location: structure-invariant direct methods
Least-squares matrix: full	Secondary atom site location: difference Fourier map
*R*[*F*^2^ > 2σ(*F*^2^)] = 0.048	Hydrogen site location: inferred from neighbouring sites
*wR*(*F*^2^) = 0.158	H atoms treated by a mixture of independent and constrained refinement
*S* = 1.21	*w* = 1/[σ^2^(*F*_o_^2^) + (0.0508*P*)^2^ + 1.9491*P*] where *P* = (*F*_o_^2^ + 2*F*_c_^2^)/3
5384 reflections	(Δ/σ)_max_ < 0.001
181 parameters	Δρ_max_ = 0.82 e Å^−^^3^
16 restraints	Δρ_min_ = −0.68 e Å^−^^3^

### Special details

**Table:**

Geometry. All esds (except the esd in the dihedral angle between two l.s. planes) are estimated using the full covariance matrix. The cell esds are taken into account individually in the estimation of esds in distances, angles and torsion angles; correlations between esds in cell parameters are only used when they are defined by crystal symmetry. An approximate (isotropic) treatment of cell esds is used for estimating esds involving l.s. planes.
Refinement. Refinement of *F*^2^ against ALL reflections. The weighted *R*-factor wR and goodness of fit *S* are based on *F*^2^, conventional *R*-factors *R* are based on *F*, with *F* set to zero for negative *F*^2^. The threshold expression of *F*^2^ > σ(*F*^2^) is used only for calculating *R*-factors(gt) etc. and is not relevant to the choice of reflections for refinement. *R*-factors based on *F*^2^ are statistically about twice as large as those based on *F*, and *R*- factors based on ALL data will be even larger.

### Fractional atomic coordinates and isotropic or equivalent isotropic displacement parameters (Å^2^)

**Table:**

	*x*	*y*	*z*	*U*_iso_*/*U*_eq_	
O1	0.12341 (16)	0.48227 (11)	0.6812 (3)	0.0260 (4)	
H1	0.181 (4)	0.502 (3)	0.635 (6)	0.039*	
O2	0.25104 (13)	0.34866 (10)	0.7264 (2)	0.0180 (3)	
O3	0.13920 (15)	0.40992 (11)	1.0319 (2)	0.0198 (3)	
O4	0.02645 (13)	0.34042 (10)	0.7334 (3)	0.0190 (3)	
O5	−0.41866 (14)	0.29289 (11)	0.4731 (3)	0.0209 (3)	
H5	−0.368 (3)	0.259 (3)	0.529 (6)	0.031*	
O6	−0.47633 (13)	0.40326 (10)	0.2142 (2)	0.0174 (3)	
O7	−0.30247 (15)	0.43252 (11)	0.4549 (2)	0.0209 (3)	
O8	−0.28270 (13)	0.32102 (10)	0.1683 (2)	0.0182 (3)	
P1	0.13662 (4)	0.39195 (3)	0.79806 (7)	0.01228 (10)	
P2	−0.36645 (4)	0.36515 (3)	0.32105 (7)	0.01211 (10)	
N11	0.03108 (16)	0.66864 (12)	0.7030 (3)	0.0185 (3)	
H11A	0.009 (3)	0.7219 (13)	0.720 (5)	0.028*	
H11B	0.1058 (17)	0.667 (2)	0.730 (5)	0.028*	
H11C	−0.017 (3)	0.638 (2)	0.780 (5)	0.028*	
H11D	0.018 (3)	0.658 (2)	0.570 (3)	0.028*	
N12	0.47645 (17)	0.41255 (13)	0.7925 (3)	0.0188 (3)	
H12A	0.500 (3)	0.402 (2)	0.920 (3)	0.028*	
H12B	0.523 (3)	0.382 (2)	0.711 (5)	0.028*	
H12C	0.479 (3)	0.4675 (13)	0.760 (5)	0.028*	
H12D	0.4031 (19)	0.395 (2)	0.770 (5)	0.028*	
N13	0.29995 (16)	0.32905 (12)	1.2991 (3)	0.0182 (3)	
H13A	0.252 (3)	0.351 (2)	1.207 (4)	0.027*	
H13B	0.285 (3)	0.339 (2)	1.428 (3)	0.027*	
H13C	0.3748 (18)	0.345 (2)	1.278 (5)	0.027*	
H13D	0.288 (3)	0.2742 (12)	1.282 (5)	0.027*	
N14	−0.18493 (17)	0.42049 (12)	0.8356 (3)	0.0178 (3)	
H14A	−0.227 (3)	0.425 (2)	0.720 (4)	0.027*	
H14B	−0.221 (3)	0.394 (2)	0.933 (4)	0.027*	
H14C	−0.169 (3)	0.4726 (14)	0.877 (5)	0.027*	
H14D	−0.1132 (19)	0.398 (2)	0.814 (5)	0.027*	

### Atomic displacement parameters (Å^2^)

**Table:**

	*U*^11^	*U*^22^	*U*^33^	*U*^12^	*U*^13^	*U*^23^
O1	0.0185 (7)	0.0193 (7)	0.0403 (10)	0.0021 (6)	0.0007 (6)	0.0145 (7)
O2	0.0140 (6)	0.0182 (6)	0.0220 (7)	0.0022 (5)	0.0025 (5)	−0.0015 (5)
O3	0.0225 (7)	0.0215 (7)	0.0153 (6)	0.0000 (5)	−0.0005 (5)	−0.0035 (5)
O4	0.0142 (6)	0.0179 (6)	0.0249 (7)	−0.0037 (5)	−0.0022 (5)	−0.0034 (5)
O5	0.0183 (6)	0.0207 (7)	0.0237 (7)	0.0025 (5)	0.0055 (5)	0.0099 (5)
O6	0.0145 (6)	0.0189 (6)	0.0188 (6)	0.0015 (5)	−0.0032 (5)	0.0032 (5)
O7	0.0221 (7)	0.0242 (7)	0.0165 (6)	−0.0048 (6)	−0.0035 (5)	−0.0031 (5)
O8	0.0150 (6)	0.0206 (7)	0.0192 (6)	−0.0010 (5)	0.0046 (5)	−0.0033 (5)
P1	0.01141 (19)	0.01162 (19)	0.01380 (19)	−0.00003 (14)	−0.00053 (14)	−0.00007 (14)
P2	0.01113 (18)	0.0140 (2)	0.01122 (18)	−0.00061 (14)	0.00038 (13)	0.00073 (14)
N11	0.0167 (7)	0.0184 (7)	0.0205 (8)	0.0015 (6)	−0.0007 (6)	−0.0001 (6)
N12	0.0170 (7)	0.0226 (8)	0.0169 (7)	0.0013 (6)	−0.0008 (6)	−0.0018 (6)
N13	0.0160 (7)	0.0199 (7)	0.0187 (7)	−0.0015 (6)	−0.0001 (5)	0.0002 (6)
N14	0.0178 (7)	0.0197 (7)	0.0159 (7)	−0.0006 (6)	0.0001 (5)	−0.0016 (6)

### Geometric parameters (Å, °)

**Table:**

O1—P1	1.5821 (17)	N11—H11D	0.877 (18)
O1—H1	0.78 (4)	N12—H12A	0.869 (18)
O2—P1	1.5287 (15)	N12—H12B	0.878 (18)
O3—P1	1.5257 (16)	N12—H12C	0.868 (18)
O4—P1	1.5262 (15)	N12—H12D	0.879 (18)
O5—P2	1.5955 (16)	N13—H13A	0.863 (18)
O5—H5	0.85 (4)	N13—H13B	0.862 (18)
O6—P2	1.5254 (15)	N13—H13C	0.892 (18)
O7—P2	1.5201 (16)	N13—H13D	0.859 (18)
O8—P2	1.5295 (15)	N14—H14A	0.874 (18)
N11—H11A	0.862 (18)	N14—H14B	0.856 (18)
N11—H11B	0.859 (18)	N14—H14C	0.861 (18)
N11—H11C	0.876 (18)	N14—H14D	0.890 (18)
			
P1—O1—H1	116 (3)	H11C—N11—H11D	110 (3)
P2—O5—H5	115 (3)	H12A—N12—H12B	107 (3)
O3—P1—O4	111.44 (9)	H12A—N12—H12C	113 (3)
O3—P1—O2	111.70 (9)	H12B—N12—H12C	111 (3)
O4—P1—O2	112.43 (9)	H12A—N12—H12D	112 (3)
O3—P1—O1	107.95 (10)	H12B—N12—H12D	108 (3)
O4—P1—O1	104.71 (10)	H12C—N12—H12D	106 (3)
O2—P1—O1	108.21 (9)	H13A—N13—H13B	118 (3)
O7—P2—O6	111.73 (9)	H13A—N13—H13C	112 (3)
O7—P2—O8	111.74 (9)	H13B—N13—H13C	107 (3)
O6—P2—O8	112.79 (9)	H13A—N13—H13D	101 (3)
O7—P2—O5	107.68 (9)	H13B—N13—H13D	105 (3)
O6—P2—O5	103.69 (9)	H13C—N13—H13D	113 (3)
O8—P2—O5	108.73 (9)	H14A—N14—H14B	114 (3)
H11A—N11—H11B	107 (3)	H14A—N14—H14C	107 (3)
H11A—N11—H11C	105 (3)	H14B—N14—H14C	109 (3)
H11B—N11—H11C	119 (3)	H14A—N14—H14D	112 (3)
H11A—N11—H11D	105 (3)	H14B—N14—H14D	112 (3)
H11B—N11—H11D	110 (3)	H14C—N14—H14D	102 (3)

### Hydrogen-bond geometry (Å, °)

**Table:**

*D*—H···*A*	*D*—H	H···*A*	*D*···*A*	*D*—H···*A*
O1—H1···O7^i^	0.78 (4)	1.80 (4)	2.570 (2)	168 (4)
O5—H5···O8^ii^	0.85 (4)	1.79 (4)	2.632 (2)	170 (4)
N11—H11A···O4^iii^	0.86 (2)	1.89 (2)	2.747 (2)	175 (4)
N11—H11B···O8^i^	0.86 (2)	2.10 (2)	2.951 (2)	171 (3)
N11—H11C···O3^iv^	0.88 (2)	1.99 (2)	2.852 (3)	169 (3)
N11—H11D···O4^i^	0.88 (2)	2.01 (2)	2.870 (2)	167 (3)
N12—H12A···O6^v^	0.87 (2)	1.91 (2)	2.755 (2)	165 (3)
N12—H12B···O5^vi^	0.88 (2)	2.16 (2)	3.008 (3)	161 (3)
N12—H12C···O6^i^	0.87 (2)	1.99 (2)	2.827 (2)	161 (3)
N12—H12D···O2	0.88 (2)	1.88 (2)	2.754 (2)	175 (3)
N13—H13A···O3	0.86 (2)	1.92 (2)	2.773 (2)	172 (3)
N13—H13B···O2^vii^	0.86 (2)	1.96 (2)	2.822 (2)	175 (3)
N13—H13C···O6^v^	0.89 (2)	1.95 (2)	2.830 (2)	168 (3)
N13—H13D···O2^ii^	0.86 (2)	1.96 (2)	2.820 (2)	176 (3)
N14—H14A···O7	0.87 (2)	1.90 (2)	2.771 (2)	174 (3)
N14—H14B···O8^vii^	0.86 (2)	2.01 (2)	2.859 (2)	171 (3)
N14—H14C···O3^iv^	0.86 (2)	1.92 (2)	2.784 (2)	178 (3)
N14—H14D···O4	0.89 (2)	1.89 (2)	2.771 (2)	171 (3)

Symmetry codes: (i) −*x*, −*y*+1, −*z*+1; (ii) *x*, −*y*+1/2, *z*+1/2; (iii) −*x*, *y*+1/2, −*z*+3/2; (iv) −*x*, −*y*+1, −*z*+2; (v) *x*+1, *y*, *z*+1; (vi) *x*+1, *y*, *z*; (vii) *x*, *y*, *z*+1.
